# RETRACTED: Mujeeb Rahman, K.K.; Subashini, M.M. Identification of Autism in Children Using Static Facial Features and Deep Neural Networks. *Brain Sci.* 2022, *12*, 94

**DOI:** 10.3390/brainsci16101127

**Published:** 2026-10-19

**Authors:** K. K. Mujeeb Rahman, M. Monica Subashini

**Affiliations:** 1 School of Electronics Engineering, Vellore Institute of Technology, Vellore 632014, India; m.rahman@ajman.ac.ae; 2 Department of Biomedical Engineering, Ajman University, Ajman P.O. Box 346, United Arab Emirates; 3 School of Electrical Engineering, Vellore Institute of Technology, Vellore 632014, India

The journal retracts the article “Identification of Autism in Children Using Static Facial Features and Deep Neural Networks” [1], which is cited above.

Following publication, concerns were brought to the attention of the Editorial Office regarding the validity of the underlying dataset used in this article.

In accordance with the journal’s standard procedure, the Editorial Office and Editorial Board conducted an investigation, which verified the concerns related to the methodology and reliability of the third-party dataset utilized in this study. The investigation was also unable to verify that the facial image dataset had been collected with appropriate ethical approval, or that informed consent had been obtained from the legal guardians of the children whose images were included in the dataset.

As the Editorial Office and Editorial Board have determined that the dataset is essential to the findings, they therefore have decided to retract and remove this article, as per MDPI’s retraction policy. This article has been removed in order to protect the identities of those included in the dataset. To maintain the integrity of the scholarly record, the title, author information and this retraction notice will remain permanently available, while the article itself has been removed. This information has also been communicated to relevant indexing services.

This retraction was approved by the Editor-in-Chief of the journal *Brain Sciences*.

The authors have been informed of this retraction and have indicated their disagreement with this decision.
